# Structure-Dependent Immune Modulatory Activity of Protegrin-1 Analogs

**DOI:** 10.3390/antibiotics3040694

**Published:** 2014-11-27

**Authors:** Susu M. Zughaier, Pavel Svoboda, Jan Pohl

**Affiliations:** 1 Department of Microbiology and Immunology, Emory University School of Medicine, Atlanta, GA 30322, USA; 2 Laboratories of Microbial Pathogenesis, Atlanta Department of Veterans Affairs Medical Center, Atlanta, GA 30033, USA; 3 Microchemical and Proteomics Facility, Emory University School of Medicine, Atlanta, GA 30322, USA; E-Mails: hug6@cdc.gov (P.S.); hhe7@cdc.gov (J.P.); 4 Biotechnology Core Facility Branch, Division of Scientific Resources, Centers for Disease Control and Prevention, Atlanta, GA 30333, USA

**Keywords:** protegrin-1 (PG-1), Toll-like receptor (TLR) ligands, cytokines, macrophage, innate immunity

## Abstract

Protegrins are porcine antimicrobial peptides (AMPs) that belong to the cathelicidin family of host defense peptides. Protegrin-1 (PG-1), the most investigated member of the protegrin family, is an arginine-rich peptide consisting of 18 amino acid residues, its main chain adopting a β-hairpin structure that is linked by two disulfide bridges. We report on the immune modulatory activity of PG-1 and its analogs in neutralizing bacterial endotoxin and capsular polysaccharides, consequently inhibiting inflammatory mediators’ release from macrophages. We demonstrate that the β-hairpin structure motif stabilized with at least one disulfide bridge is a prerequisite for the immune modulatory activity of this type of AMP.

## 1. Introduction

The innate immune system protects the host by rapid detection and elimination of invading pathogens. Phagocytic cells are equipped with pattern recognition receptors (PRR) such as Toll-like receptors (TLRs) [1], scavenger receptors, and others that facilitate rapid detection of invading pathogens [2,3]. Phagocytes are also equipped with bactericidal compounds like lysozymes and host defense cationic peptides that facilitate rapid killing of pathogens [4,5,6]. Antimicrobial peptides (AMPs) are ubiquitous in many host cells and found as preformed structures stored in granules inside the immune cells that can be instantly released and activated [7,8,9,10]. AMPs are also induced and synthesized during infection, which helps increase their level to augment host defense [7,8,9,10,11].

Upon bacterial infection, one of the proposed mechanisms of antibacterial activity of AMPs is insertion into bacterial membranes, causing the rupture and death of bacteria. The ruptured bacterial membrane fragments and leaked cytosol contents contain potent pathogen-associated molecular patterns (PAMPs) that activate Toll-like receptors (TLRs), leading to the release of inflammatory mediators [12,13]. However, the excessive release of inflammatory mediators also causes uncontrolled immune activation and sepsis-like symptoms [14,15,16]. Therefore, AMPs play an important role in dampening the acute release of proinflammatory mediators by binding to pathogen-related TLR ligands and inhibiting their bioactivity and/or, for some AMPs, by directly intervening in the TLR signaling cascade [17,18,19,20]. Endotoxin, also known as lipopolysaccharide (LPS) or lipooligosaccharide (LOS), is a major component of the outer membrane in Gram-negative bacteria. The direct interaction of AMPs with LPS is well documented and underlies the neutralizing activity or the immune modulatory effects *in vitro* and *in vivo* [17,21]. We observed that while AMPs dampen proinflammatory cytokine release induced by LPS, they also amplify respiratory burst in macrophages, possibly to ensure the killing of invading pathogens [22]. The important role of AMPs in host defense is due to their ability to exert both antibacterial activity and immune modulatory activity on host cells [23,24,25]. This dual role of AMPs is important for clearing invading pathogens and resolving subsequent inflammation [26].

**Figure 1:**
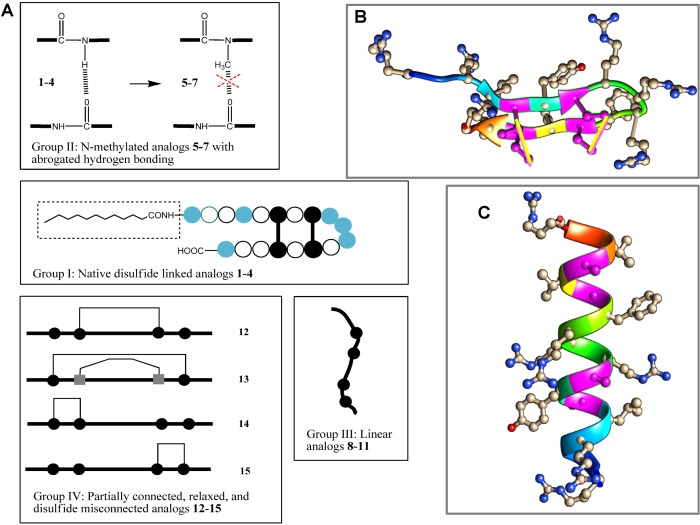
PG-1 and its analogs used in this study. (**A**) Schematic overview of analogs. [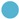 = arginine or homoarginine (**4**); 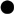 = cysteine or 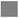 homocysteine (**13**); absence of H-bonding in **5**–**7** is indicated (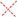)]; the dotted box indicates the acyl chain in analog **3**. Three-dimensional structure model of PG-1 (**B**) and of its linearized non-cysteine containing analog **8** (**C**) with alanine replacements. The 3D structure of the PG-1 β-hairpin fold is predicted by I-TASSER based on the published crystal structures (PDB # 1PG1 and 1ZY6) and visualized using Chimera software. Cysteine residues that form disulfide bridges are magenta-colored. The 3D structure of a linearized analog **8** adopting a coil fold is achieved when four alanine residues are replaced by cysteines. The coil fold is predicted by I-TASSER and visualized by Chimera. Alanine residues are magenta-colored.

Protegrins are the main porcine AMPs that belong to the cathelicidin family of host defense peptides and consist of five members, PG-1, 2, 3, 4, and 5 [27,28]. PG-1 is the most abundant and most characterized member of the protegrins. PG-1 is an arginine-rich AMP that consists of 18 amino acids, including four cysteines; its main chain adopts a β-hairpin structure that is linked with two disulfide bridges (Figure 1) [29,30,31]. PG-1 is assumed to exert its antibacterial activity by forming β-barrel pores across the phospholipid membranes, leading to cell death [32]. PG-1 forms dimers that when inserted into the bacterial membranes build octameric transmembrane pores, causing major leak of potassium ions and subsequent cell rupture, as shown by the molecular dynamic simulation studied by Kaznessis [33]. Due to its cationic character, PG-1 acts rapidly by binding electrostatically to anionic bacterial lipid membranes [34,35,36]. PG-1, like other AMPs, possesses potent antibiotic-like activity and avoids antibiotic resistance systems due to its rapid, nonspecific effect on bacterial cell membranes [37,38]. It was estimated that between 10 and 100 pores per bacterial cell are required to exert a bactericidal effect on *E. coli* [39].

While several studies have extensively investigated the structural determinants of PG-1 antibacterial activity [20,39,40,41,42,43,44,45], the structural determinants of PG-1 required for immune modulatory activity are not known. In the search for PG-1 analogs that exhibit enhanced bactericidal activity with reduced toxicity, several synthetic PG-1 analogs have been reported [27,28,46,47,48,49,50,51,52]. The major structural characteristics of PG-1 peptide required for antibacterial activity include: (a) β-hairpin fold stabilized with two disulfide bridges connecting Cys-6 and Cys-15 and Cys-8 and Cys-13, respectively, and intra-chain hydrogen bonding; (b) cationic nature; and (c) amphipathicity of the peptide [38,39]. Synthetic analogs containing the two disulfide bridges are more active than analogs containing a single or no disulfide bridge [38,53,54]. The increased cationic character of the synthetic peptide due to an increased number of arginine residues resulted in enhanced antibacterial activity [55]. PG-1, as a cationic peptide, has the ability to bind to anionic components of bacterial membranes; this includes LPS and capsular polysaccharide (CPS) polymers which act as Toll-like receptor (TLR) ligands and thus function as potent inducers of inflammatory responses in macrophages [56,57].

In this study we investigated the immune modulatory activity of PG-1 and several of its analogs and evaluated the importance of disulfide bridges as well as hydrogen bonding potential of its main chain on the ability to neutralize TLR ligand bioactivity in macrophages, consequently dampening inflammatory mediators’ release. We report that synthetic PG-1 analogs adopting (and maintaining) the β-hairpin fold bearing at least one disulfide bridge exert potent immune modulatory activity against meningococcal LOS as TLR4 ligand and CPS polymers as TLR2 and TLR4 ligand.

## 2. Results and Discussion

As a first line of innate host defense against invading pathogens, AMPs can exert significant immune modulatory activity on mammalian cells [58]. In this respect, human alpha-helical LL-37 cathelicidin has been extensively studied [17,21,57,59,60]. We previously reported that porcine cathelicidin, PG-1, inhibits meningococcal LOS immune stimulatory activity and reduces TNFα and nitric oxide release from human and murine macrophages, respectively [22], indicating that a similar mechanism applies to this beta-forming peptide. Herein, we extended our investigations to several analogs of PG-1 (Figure 1 and Table 1) and tested their immune modulatory activity against TLR ligand endotoxin and capsular polysaccharides CPS prepared from *Neisseria meningitidis*. To investigate the immune modulatory activity of the PG-1 analogs, we employed human and murine macrophage cell lines stimulated with TLR ligands that have been preincubated with these peptides (2 µg/mL, a physiologically relevant dose). The immune modulatory activity was assessed as the inhibition of proinflammatory cytokines TNFα and IL-1β released from stimulated human THP-1 monocyte-like macrophages [57,61]. Inhibition of nitric oxide release from murine RAW264 macrophages was also used to assess the immune modulatory activity of the derivatives. All analogs were non-toxic when used at this low dose (2 µg/mL per 10^6^ macrophages) and did not affect the viability of macrophages as assessed by the trypan blue exclusion method [62] (data not shown). None of the PG-1 analogs tested in this study induced the release of cytokines TNFα and IL-1β or nitric oxide when added to macrophages without TLR ligands.

**Figure 2:**
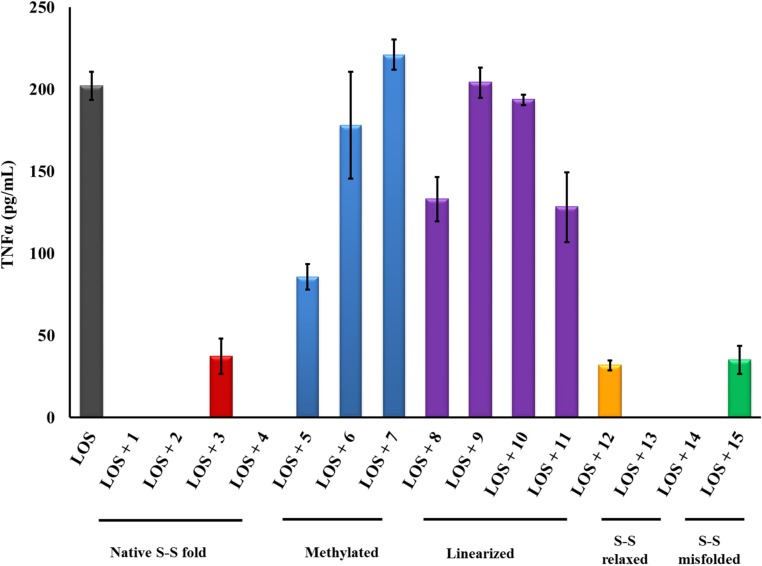
PG-1 analogs neutralized meningococcal LOS activity and inhibited TNFα release. TNFα was released from human macrophage-like THP-1 cells induced overnight with meningococcal LOS (5 ng/mL ~ 2.5 pmole/mL) preincubated with or without 2 µg/mL of PG-1 or its derivatives for 30 min at 37 °C. TNFα release was measured by ELISA. Error bars represent ±SD from the mean of duplicate measurements. This experiment is representative of two independent experiments. Methylated: N-methylated tyrosine or phenylalanine derivatives; S-S: disulfide bridges.

**Figure 3:**
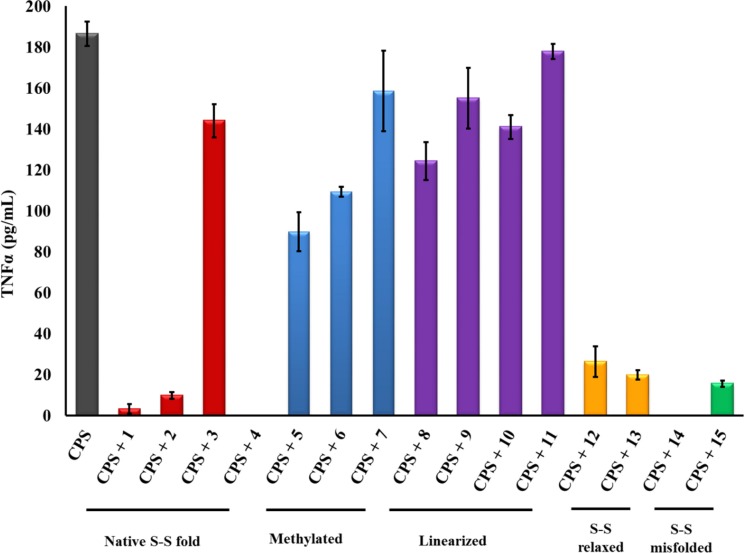
PG-1 analogs neutralized meningococcal capsular polysaccharide (CPS) polymer activity and inhibited TNFα release. CPS polymers were purified from the endotoxin-deficient serogroup B meningococcal NMB-*lpxA* mutant designated CPS. TNFα was released from human macrophage-like THP-1 cells induced overnight with meningococcal CPS polymers (25 µg/mL) pre-incubated with or without 2 µg/mL of PG-1 or its derivatives for 30 min at 37 °C. TNFα release was measured by ELISA. Error bars represent ±SD from the mean of duplicate measurements. This experiment is representative of two independent experiments. Methylated: N-methylated tyrosine or phenylalanine derivatives; S–S: disulfide bridges.

**Figure 4:**
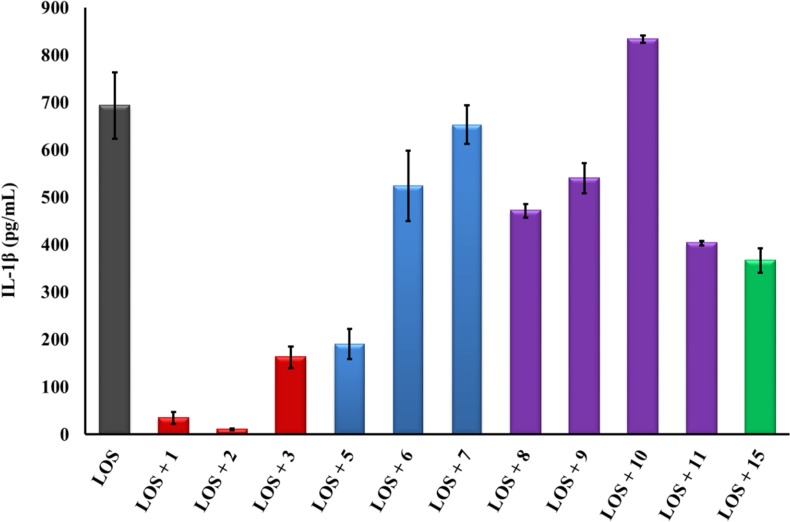
PG-1 analogs neutralized meningococcal LOS activity and inhibited IL-1β release. IL-1β was released from human macrophage-like THP-1 cells induced overnight with meningococcal LOS (5 ng/mL ~ 2.5 pmole/mL) preincubated with or without 2 µg/mL of PG-1 or its derivatives for 30 min at 37 °C. IL-1β release was measured by ELISA. Error bars represent ±SD from the mean of duplicate measurements. This experiment is representative of two independent experiments. Methylated: N-methylated tyrosine or phenylalanine derivatives; S-S: disulfide bridges.

**Figure 5:**
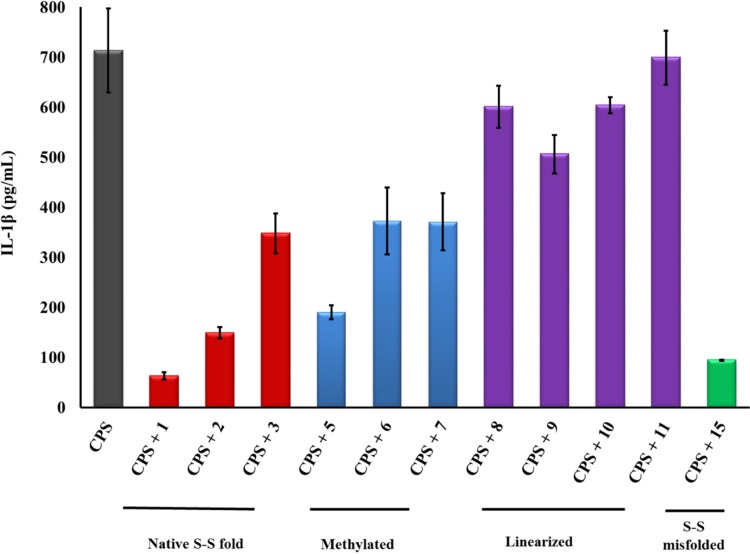
PG-1 analogs neutralized meningococcal capsular polysaccharide (CPS) polymers activity and inhibited IL-1β release. CPS polymers were purified from the endotoxin-deficient serogroup B meningococcal NMB-*lpxA* mutant designated CPS. IL-1β was released from human macrophage-like THP-1 cells induced overnight with meningococcal CPS polymers (25 µg/mL) pre-incubated with or without 2 µg/mL of PG-1 or its derivatives for 30 min at 37 °C. IL-1β release was measured by ELISA. Error bars represent ±SD from the mean of duplicate measurements. This experiment is representative of two independent experiments. Methylated: N-methylated tyrosine or phenylalanine derivatives; S–S: disulfide bridges.

**Figure 6:**
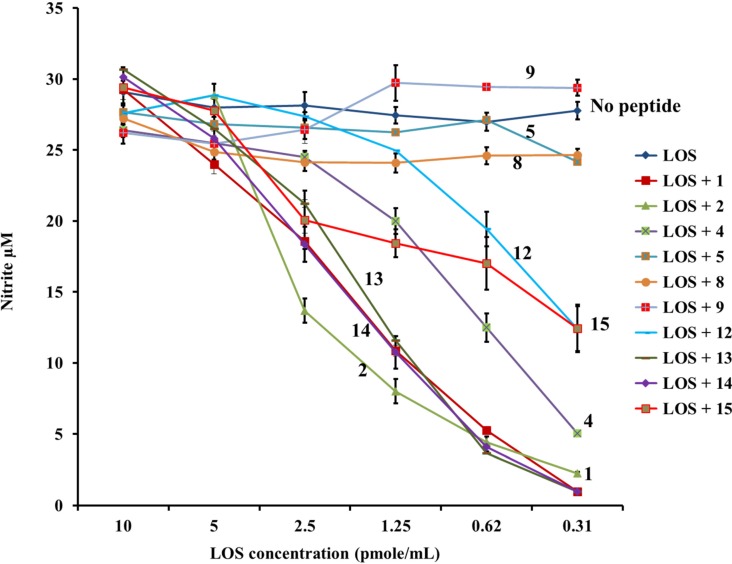
PG-1 analogs neutralized meningococcal LOS bioactivity and inhibited nitric oxide release. Nitric oxide was released from murine RAW264 macrophages induced overnight with meningococcal LOS doses pre-incubated with or without 2 µg/mL of PG-1 or its analogs for 30 min at 37 °C. Nitric oxide release was measured by the Greiss method. Error bars represent ± SD from the mean of duplicate measurements. This experiment is representative of two independent experiments.

**Figure 7:**
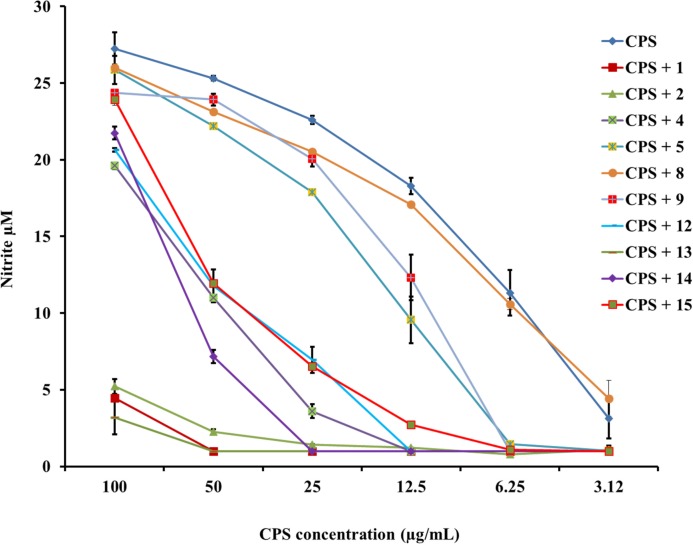
PG-1 analogs neutralized meningococcal capsular polysaccharide (CPS) polymer activity and inhibited nitric oxide release. CPS polymers were purified from the endotoxin-deficient serogroup B meningococcal NMB-*lpxA* mutant designated CPS. Nitric oxide was released from murine RAW264 macrophages induced overnight with doses of meningococcal CPS polymers pre-incubated with or without 2 µg/mL of PG-1 or its analogs for 30 min at 37 °C. Nitric oxide release was measured by the Greiss method. Error bars represent ±SD from the mean of duplicate measurements. This experiment is representative of two independent experiments.

Specifically, we investigated the immune modulatory response towards completely or partially linearized analogs, testing the importance of disulfide bridges of PG-1 (Compound **1**). We also tested the importance of intra-/inter-chain hydrogen bonding potential, as H-bonding is presumed to be a major driving force behind oligomerization of PG-1 at bacterial membranes [63]. The analogs used in this work are categorized into four groups based on their common structural characteristics (Figure 1 and Table 1). Group I derivatives **1**–**4** include the native PG-1 (**1**) and all maintain disulfide bridge connectivity. As expected, the D-amino acid analog **2** has very similar activity to the parent peptide **1** and effectively neutralized meningococcal LOS and CPS bioactivity, leading to inhibition of TNFα (Figure 2 and Figure 3), IL-1β (Figure 4 and Figure 5), and nitric oxide (Figure 6 and Figure 7) release even when used at low dose of 2 µg/mL [52]. Peptide **3** contains a dodecanoyl (C12) moiety at the N-terminus of **1**, making it more hydrophobic. Compared to PG-1, acylation did not improve immune modulatory activity against meningococcal LOS (Figure 2 and Figure 4) and was even less effective against neutralizing CPS polymers (Figure 3 and Figure 5). This observation is contrary to LL-37, for which N^α^-terminus acylation markedly enhanced its immune modulatory activity against LOS and CPS polymers [57]. Furthermore, acylation of the cathepsin G (CG) peptide, which has an α-helical structure, led to enhancement of its activity [64,65]. The length of fatty acyl chain matters and C12 chain length were found to be optimal for enhancing CG peptide activity when compared to shorter or longer chain lengths ranging from C4–C18, as previously shown [66]. Therefore, acylation of peptides with a β-hairpin structure like PG-1 may potentially interfere with peptide oligomerization, which is a very refined process prerequisite to its activity [32]. Analog **4** contains in place of its six arginine residues homoarginine residues, thus making it more apolar as compared to the parent peptide **1** while retaining the positively-charged guanidinium groups. Side-chain guanidinium groups of Arg residues were shown to be critical for PG-1 interaction with bacterial surface phosphate moieties [67,68]. It can be seen that the replacement of arginines with bulkier homoarginines resulted in immune modulatory derivative **4**, the most potent that we have tested so far against meningococcal LOS and CPS (Figure 2, Figure 3, Figure 6, and Figure 7). We note that the number of arginines was found to be critical for protegrin antimicrobial activity [69]. Tang *et al*. showed that reducing the number of arginines dramatically reduced antibacterial activity due to a reduction in membrane insertion and the inability of arginine to electrostatically bind to the phosphate groups on lipid A [55,69,70]. The presence of phosphate groups is critical for interaction with cationic peptides, and elimination of these negative charges by phosphoethanolamine or carbohydrate residues replacement confers resistance to AMPs like polymyxin B and LL-37 [71]. PG-1 is also shown to bind more efficiently to LPS from *Pseudomonas aeruginosa* as compared to LPS from *Burkholderia cepacia* [72]. The reduced binding due to substitution of phosphate head groups with 4-aminoarabinose in *B. cepacia* lipid A is thought to be the major determinant of resistance [72].

**Table 1:** PG-1 and its analogs used in this study and their activities.

Comp-ound	Sequence										LOS activity inhibition (%)	CPS activity inhibition (%)
	1 2 3 4 5	6	7	8	9 10 11	12	13	14	15	16 17 18		
1 (I)	NH_2_-R G G R L	C	Y	C	R R R	F	C	V	C	V G R-CONH_2_	99.9	99
2 (I)	NH_2_-*R G G R L*	*C*	*Y*	*C*	*R R R*	F	*C*	*V*	*C*	*V G R*-CONH_2_	98.3	94.7
3 (I)	C12-R G G R L	C	Y	C	R R R	F	C	V	C	V G R-CONH_2_	74.2	22.5
4 (I)	NH_2_-R G G R L	C	Y	C	R R R	F	C	V	C	V G R-CONH_2_	100	100
5 (II)	NH_2_-R G G R L	C	Y	C	R R R	F	C	V	C	V G R-CONH_2_	60	52
6 (II)	NH_2_-R G G R L	C	Y	C	R R R	F	C	V	C	V G R-CONH_2_	16	21
7 (II)	NH_2_-R G G R L	C	Y	C	R R R	F	C	V	C	V G R-CONH_2_	0	15
8 (III)	NH_2_-R G G R L	A	Y	A	R R R	F	A	V	A	V G R-CONH_2_	33	23
9 (III)	NH_2_-R G G R L	C(Me)	Y	C(Me)	R R R	F	C(Me)	V	C(Me)	V G R-CONH_2_	0	5.7
10 (III)	NH_2_-R G G R L	M	Y	M	R R R	F	M	V	M	V G R-CONH_2_	4.6	24
11 (III)	NH_2_-R G G R L	M(O)	Y	M(O)	R R R	F	M(O)	V	M(O)	V G R-CONH_2_	20.5	4.6
12 (IV)	NH_2_-R G G R L	C(Me)	Y	C	R R R	F	C	V	C(Me)	V G R-CONH_2_	84.2	84.7
13 (IV)	NH_2_-R G G R L	C	Y	C	R R R	F	C	V	C	V G R-CONH_2_	99.9	88.4
14 (IV)	NH_2_-R G G R L	C	Y	C	R R R	F	C(Me)	V	C(Me)	V G R-CONH_2_	99.9	99.9
15 (IV)	NH_2_-R G G R L	C(Me)	Y	C(Me)	R R R	F	C	V	C	V G R-CONH_2_	88.4	91.6

d-Amino acid residues are in italics; 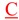: homocysteine; 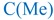: S-methyl-cysteine; C12: dodecanoyl; F: N-methyl-phenylalanine; M(O): methionine oxide; R: homoarginine; and Y: N-methyl-tyrosine.

### 2.1. β-hairpin Analogs with Impaired H-Bonding

Group II derivatives **5**–**7** maintain the native disulfide bridge connectivity but differ from **1**–**4** in having N^α^-methylated residues, Tyr-7 and/or Phe-12 in sequence positions 7 and 12. Replacement of the native N^α^-amino group in a peptide bond (-CONH-) with its N^α^-methylated surrogate, -CON(CH3)- [73], was done in order to eliminate hydrogen bonding of the main chain peptide H-bond donor (=NH) in positions 7 and/or 12 as depicted in Figure 1A. Such derivatives should exhibit weaker inter-/intra-chain H-bonding at these sites to the main chain carbonyl group acceptors that were shown to be involved in PG-1 oligomerization [74,75]. Indeed, as can be seen in Figure 2, Figure 3, Figure 4 and Figure 5, the ability of analogs **5**–**7** to neutralize meningococcal LOS and CPS was dramatically reduced in both assays. We find that the effect of N-methylation is site-specific, and more pronounced in position 7 as compared to position 12. This quantitative difference in effect can be rationalized by position 7 being in the center of the putative dimerization β-strand domain of PG-1, as opposed to position 12 at the edge of PG-1’s dimerization domain. We inferred that dimerization is important for PG-1 to have its TLR-mediated effect. This demonstrates that main chain H-bonding plays a critical role in a peptide’s binding to LOS and to CPS. In support of our findings, Giacometti *et al*. reported that synthetic PG-1 analog IB-367 neutralized LPS and led to significant reduction in TNFα levels, consequently preventing endotoxin-induced mortality in an *in vivo* rat model [20].

### 2.2. Linear PG-1 Analogs Lacking β-Hairpin

It is well established that the PG-1 peptide β-hairpin structure fold is essential for its antibacterial activity. However, it is not known whether this fold is also required for the peptide’s immune modulatory activity. To this end, the Group III analogs (**8**–**11)** represent “linearized” versions of PG-1 that do not contain native disulfide bridges of 6–15 and 8–13. We substituted the four Cys residues in analogs **8**–**11** with residues of increasing bulkiness and apolar character (Ala, Cys(S-me), Met(O), and Met, in that order). As can be seen in Table 1, “linearization” of PG-1 dramatically reduced its immune modulatory activity against meningococcal LOS and CPS (Table 1), as it failed to inhibit the release of TNFα (Figure 2 and Figure 3) or IL-1β (Figure 4 and Figure 5) from stimulated THP-1 cells. Analogs **8** and **9** also failed to inhibit nitric oxide release from murine RAW264 macrophages stimulated with LOS or CPS doses (Figure 6 and Figure 7). We also note that linearized peptides lost between 50-fold and 4000-fold of their antibacterial activity (data not shown), which is consistent with previously published reports [30,40,76]. As predicted by computational modeling of analog **8** (Figure 1C), the peptide adopts a linear coil structure rather than a β-hairpin fold native peptide structure. A previous study by Lai *et al*. designed cysteine-free linearized PG-1 analogs that adopted the β-hairpin fold by using d-proline instead of arginine at position 10, which allowed peptides to form a β-hairpin fold [77]. These linearized peptides containing d-proline maintained the β-hairpin fold and exerted good antibacterial activity [77]. It remains to be determined whether these d-proline-containing linearized peptides would also possess immune modulatory activity. The binding of PG-1 to LPS inhibits the biological activity of LPS and prevents it from activating TLR4, thus inhibiting the consequent release of cytokines TNFα and IL-1β from macrophages. Likely, the β-hairpin fold affords a peptide conformation that facilitates binding to LPS via the negative charges of the lipid A phosphate head groups and via hydrophobic interactions with lipid A fatty acyl chains, similar to what has been proposed for other AMPs. In particular, a parallel emerges with the fish defense peptides, pardaxins, where adaptive changes in the overall peptide shape enable binding to lipid A head groups as well as to hydrophobic fatty acyl chains [78]. A similar pattern of interaction was also observed between LPS and the horseshoe crab major AMP, tachyplesin 1 [79].

### 2.3. PG-1 Analogs with Altered Disulfide Connectivity

Finally, analogs **12**–**15** of Group IV address the potential importance of a native or alternative protegrin disulfide fold in complex formation with LOS and CPS. Here we report a limited number of such derivatives: analog **13** bears a “relaxed” disulfide formed by two homocysteine residues in place of cysteines; analog **12** bears a single native disulfide, Cys-8–Cys-13, in which the non-bridged cysteines were S-protected by a methyl group. Both analogs **12** and **13** reduced TNFα release from THP-1 cells (Figure 2 and Figure 3) and nitric oxide release from RAW264 macrophages in a manner comparable to that of the parent PG-1 (Figure 6 and Figure 7). Therefore, the replacement of only two cysteines with homocysteine (double native S–S connectivity) or S-methycysteine (single S–S connectivity), which potentially leads to a more relaxed β-hairpin fold structure with one or two native folded disulfides, did not substantially reduce the peptide’s immune modulatory activity. We also report on the two analogs that bear a single disulfide that is *non*native, or “misconnected”: analog **14** with a bridge connecting Cys-6 and Cys-8, and analog **15** with a bridge connecting Cys-13 and Cys-15 (Figure 1A). Surprisingly, we found that the misconnected disulfide bridges do not exert a deleterious effect because both analogs retain potent immune modulatory activity comparable to parent peptide **1**. Both analogs **14** and **15** neutralized meningococcal LOS and CPS activity and inhibited TNFα (Figure 2 and Figure 3) release from human THP-1 cells and nitric oxide release from murine RAW264 macrophages stimulated with doses of meningococcal LOS (Figure 6) or CPS (Figure 7). Analog **15** also inhibited IL-1β release from THP-1 cells (Figure 4 and Figure 5). Taken together, the data suggest that disulfide bridge alteration and/or misconnection does not impair immune modulatory activity. We therefore assume that the active β-hairpin fold can still be maintained by these modified peptides.

The ability of AMPs to exert both antibacterial and immune modulatory effects points to their therapeutic potential. LPS released from gut microbiota circulating in blood and tissues due to increased permeability of the gut mucosa can cause inflammation [80,81,82]. This microbial translocation is associated with immune activation and inflammation in chronic diseases like HIV infection [83] and chronic kidney disease [84]. Host-derived cationic peptides bind to PAMPs and neutralize their immune stimulatory activity, thereby reducing immune activation and inflammatory state.

In summary, porcine PG-1 is a very potent immune-modulatory AMP capable of complex formation with a wide range of bacterial lipopolysaccharides as well as negatively charged capsular polysaccharides. We demonstrated its ability to effectively dampen major inflammatory signaling pathways such as those of the host during the course of infection. This is the first attempt to characterize the structure-immunomodulatory relations of PG-1, a short disulfide-linked AMP. Our results suggest that the immunomodulatory activity of PG-1 is more tolerant to major structural alterations as compared to its AMP activity. This includes retaining activity while one of the native disulfides is missing and/or is misconnected. Retaining one disulfide is, however, essential as linear analogs proved inactive. Importantly, the elimination of two hydrogen (H)-bonding sites, believed to be important in the oligomerization of PG-1 on bacterial surfaces via main chain modification (methylation), proved to abrogate immunomodulatory activity of the peptide, suggesting that its active LPS-complexed form is an oligomer similar to PG-1’s AMP action. With this in mind, studies are underway to characterize the solution structures of some of these analogs in order to shed more light on this interesting phenomenon. Although statistical analysis was not performed on our peptide screening data, the number of technical replicates does suggest trends. We recognize that further work is required to test the conclusions drawn from this proof-of-concept study. Thus, future experiments are planned to further investigate the most promising peptides that exhibit potent immune modulatory activity.

## 3. Experimental Section

### 3.1. Reagents

RPMI 1640 medium, Dulbecco’s Eagle medium, fetal bovine serum (FBS), penicillin/streptomycin, sodium pyruvate, and nonessential amino acids were obtained from Cellgro Mediatech (Herndon, VA, USA). Human and mouse TNFα and IL-1β ELISA kits were from R&D Systems (Minneapolis, MN, USA). THP-1 and RAW264 cell lines were purchased from ATCC (Manassas, VA, USA). Meningococcal lipooligosaccharides (LOS/LPS) that activate TLR4 and meningococcal capsular polysaccharides (CPS) polymers that induce TLR2 and TLR4 signaling were prepared as previously described [56]. CPS polymers were purified from the LPS-deficient serogroup B *Neisseria meningitidis lpxA* mutant [56].

### 3.2. PG-1 Analog Synthesis

The PG-1 and its analogs (Table 1) used in this study were prepared by Fmoc/tBu solid-phase peptide synthesis, as previously described [85]. Fmoc-Cys(Trt)-OH and Fmoc-Hcy(Trt)-OH were used for incorporation of Cys and Hcy. Following deprotection/cleavage in TFA, the peptides were purified as all-reduced species by preparative reversed-phase- (RP) HPLC using gradients of acetonitrile in 0.1% aqueous TFA [85]. For PG-1 analogs bearing one or two disulfides, connecting Cys or Hcy residues in PG-1 sequence positions 6, 8, 13, and 15, disulfide formation was affected by air oxidation in water in the presence of charcoal using purified, all-reduced peptides [86]. The completion of oxidation was monitored by analytical RP-HPLC and mass spectrometry. The oxidized peptides were purified by RP-HPLC and lyophilized. The final peptide purity (>95%) was confirmed by RP-HPLC and peptide masses were confirmed by mass spectrometry. All peptides were used in the form of their TFA salts. The stock solutions were prepared in 0.1% aqueous acetic acid and were ultrafiltered prior to their use. When tested for their antimicrobial activity against *Neisseria gonorrhoeae* strain FA19 [58], PG-1 (**1**) and its linearized analog (**8**) demonstrated similar potencies to those published under similar conditions [87].

### 3.3. Cell Cultures

THP-1 human monocyte-like cells were grown in RPMI 1640 with L-glutamate supplemented with 10% FBS, 50 IU/mL of penicillin, 50 µg/mL of streptomycin, 1% sodium pyruvate, and 1% non-essential amino acids. Culture flasks were incubated at 37 °C with humidity under 5% CO_2_. Murine macrophages RAW264 were grown in Dulbecco’s Eagle medium, supplemented and incubated as noted above.

### 3.4. Cellular Activation

Human THP-1 (monocyte-like cells) and murine RAW264 macrophages were stimulated with TLR ligands with or without preincubation with PG-1 and its analogs (Table 1). Purified meningococcal CPS samples were freshly dissolved in pyrogen-free sterile H_2_O at 1 mg/mL stock concentration and vortexed for 2 min. Working CPS concentrations (ranging from 100 µg/mL to 1 µg/mL) were made in duplicate wells using sterile PBS by serial fold dilutions in the 96-well tissue culture plates (Becton Dickinson, Franklin Lakes, NJ, USA) at 50 µL final volumes. PG-1 analogs (2 µg/mL) or PBS equivalent volumes were added to designated wells and preincubated for 30 min at 37 °C. Freshly grown THP-1 cells and murine macrophages, each adjusted to 10^6^ cell/mL and 250 µL aliquots, were dispensed into each well at a final cell density of 250 × 10^3^ in the designated 96-well plates. The plates were incubated overnight at 37 °C with 5% CO_2_ and humidity. Supernatants from stimulated cells were harvested and stored at −20 °C until use.

### 3.5. Cytokine Profiles

The cytokines TNFα and IL-1β, released from THP-1 cells, were quantified by DuoSet ELISA (R&D Systems), as previously described [61]. All experiments were performed twice (n = 2) with technical duplication in each experiment.

### 3.6. Nitric Oxide Induction by Murine Macrophages

Freshly grown adherent RAW264 macrophages were harvested, washed and re-suspended in Dulbecco’s complete media, counted and adjusted to 10^6^ cell/mL. Two hundred fifty microliter aliquots were then dispensed into each well of a 96-well plate at a final cell density of 250 × 10^3^ prior to stimulation with TLR ligands with or without PG-1 analogs, as mentioned above. The induced RAW264 macrophages were incubated overnight at 37 °C with 5% CO_2_ and supernatants were harvested and saved. Nitric oxide release was quantified using the Greiss chemical method, as previously described [61].

### 3.7. Cellular Viability Assessment

Trypan blue exclusion method was used to assess the viability of macrophages (1 × 10^6^/mL) incubated with 2 µg/mL of PG-1 or its analogs overnight at 37 °C with 5% CO_2_, as described above [22,62].

### 3.8. Computational Modeling of PG-1’s 3-D Structure and Its Linearized Analogs

Three-dimensional structures of the parent PG-1 peptide and its linearized analog **8** were predicted using I-TASSER [88], and the generated PDBs were visualized by Chimera software [89]. The prediction of PG-1 was based on the published crystal structures PDB # 1PG1 and 1ZY6. The following amino acid sequences were used to generate 3D structure prediction: PG-1: NH_2_-RGGRLCYCRRRFCVCVGR-CONH_2_; Compound **8**: NH_2_-RGGRLAYARRRFAVAVGR-CONH_2_.

## 4. Conclusions

Protegrin, as a major porcine leukocyte AMP, exerts potent immune modulatory activity. The data presented here suggest that adoption of the β-hairpin structure, stabilized with at least a single disulfide bridge, is a prerequisite for immune modulatory potential. Active PG-1 analogs neutralized LOS and CPS bioactivity and markedly reduced inflammatory mediators’ release from macrophages.
